# Lifestyle in Multiple Myeloma - a longitudinal cohort study protocol

**DOI:** 10.1186/s12885-016-2407-x

**Published:** 2016-07-04

**Authors:** M. Heinrich, A. Fisher, B. Paton, O. McCourt, R. J. Beeken, A. Hackshaw, J. Wardle, K. Yong

**Affiliations:** Health Behaviour Research Centre, Department of Epidemiology and Public Health, University College London, London, UK; Institute of Sport, Exercise and Health, London, UK; Cancer Institute, University College London, London, UK

**Keywords:** Multiple myeloma, cohort, physical activity, quality of life

## Abstract

**Background:**

Deterioration in bone health is one of the presenting symptoms of Multiple Myeloma (MM), a cancer of plasma cells. As a consequence of this condition, patients suffer bone pain and bone damage and report cancer-related fatigue, resulting in deterioration in their quality of life. Evidence in patients with solid tumours shows promise for the positive effects of physical activity on quality of life. However, in the case of patients with MM a better understanding of the association between physical fitness and quality of life factors is still required. Therefore, this cohort study aims to objectively and longitudinally assess activity and fitness levels in patients with MM in order to explore their role in bone health, fatigue and quality of life for this patient population.

**Methods/Design:**

The study is a prospective cohort study of MM patients in remission to assess physical activity, fatigue and bone health. Clinical markers of health, self-reported measures of psychological and physical well-being, and lifestyle behaviours are assessed at baseline, 3, 6 and 12 months. At each time point, patients complete cardiopulmonary exercise testing (CPET) along with a series of objective tests to assess physical fitness (eg accelerometry) and a number of self-report measures. A complementary qualitative study will be carried out in order to explore patients’ desire for lifestyle advice and when in their cancer journey they deem such advice to be useful.

**Discussion:**

This study will be the first to prospectively and longitudinally explore associations between physical fitness and well-being, bone health, and fatigue (along with a number of other physical and clinical outcomes) in a cohort of patients with MM with the use of objective measures. The findings will also help to identify time points within the MM pathway at which physical activity interventions may be introduced for maximum benefit.

## Background

Multiple Myeloma (MM) accounts for around 10 % of all haematological cancers [1], with approximately 5500 new cases each year in the UK [2]. It is incurable, but effective disease-directed therapies are extending life expectancy and patients often enter a long plateau phase (remission), where they require no (or only maintenance) treatment. A main presenting feature of MM is abnormal bone metabolism with around 80 % of patients demonstrating bone morbidity [3], putting them at high risk of fracture, pain and vertebral collapse, leading to skeletal deformity, muscle wasting and deconditioning [4]. Cancer-related fatigue (CRF) is another clinical feature observed in a very large proportion, with symptoms often persisting long after treatment has ceased [5]. CRF has been identified as one of the most distressing cancer symptoms, with some patients rating it even above pain [6–8] deterring patients from further treatment, impacting recovery and survival rates [9]. The aetiology of fatigue in cancer is multifactorial, including anaemia, systemic reaction to tissue injury caused by the disease, infections, sleep disturbance, psychosocial factors [5]. An emerging body of literature demonstrates that chemotherapy is extremely detrimental to health related fitness, and inefficiency of the cardiovascular system may contribute to fatigue and impair quality of life.

Given good evidence that physical activity can improve fitness, bone health and reduce fatigue in other populations (including other cancers), it is feasible that physical activity could be of particular importance in improving outcomes for patients with MM. In a retrospective study 88 MM survivors were asked to report their exercise behaviour during and post-treatment. Although this study was limited by the use of self-report and recall, activity levels were extremely low with 6.8 % and 20.4 % of participants meeting minimum activity guidelines during and off-treatment respectively [10] (which is likely an over-estimate). However, more physical activity was related to higher scores on all domains of quality of life [10]. In a qualitative study patients expressed strong desire for physical activity advice, but fear of initiating exercise was a barrier [11]. Studies using objective measures of physical activity and health-related fitness, along with clinical and patient reported outcomes, are required to increase our understanding of how activity levels and fitness change over time post-diagnosis; how these factors relate to bone health and fatigue and when and how patients would like lifestyle advice to be provided are also required before we can provide tailored exercise interventions to this unique population.

Cohort studies with a focus on physical activity and fitness in cancer survivors are limited generally, indeed to our knowledge only one study; the AMBER cohort of breast cancer patients [12], is currently collecting longitudinal data on objectively measured activity, along with clinical outcomes. Here we describe the Myeloma – Advancing Survivor Cancer OuTcomes (MASCOT) cohort study. To our knowledge, MASCOT is the first study to gather longitudinal objective data on physical activity, fitness and clinical and patient reported outcomes in multiple myeloma.

The aims of the MASCOT cohort study are to examine objectively measured activity levels (and health related fitness) at multiple time points following treatment, and to examine how activity and fitness relate to markers of bone health, fatigue and a number of clinical, physical and patient reported outcomes. This will form a comprehensive study into the benefits of physical activity on the survivorship outcomes of patients with Multiple Myeloma.

## Methods

### Study design

**Fig. 1:**
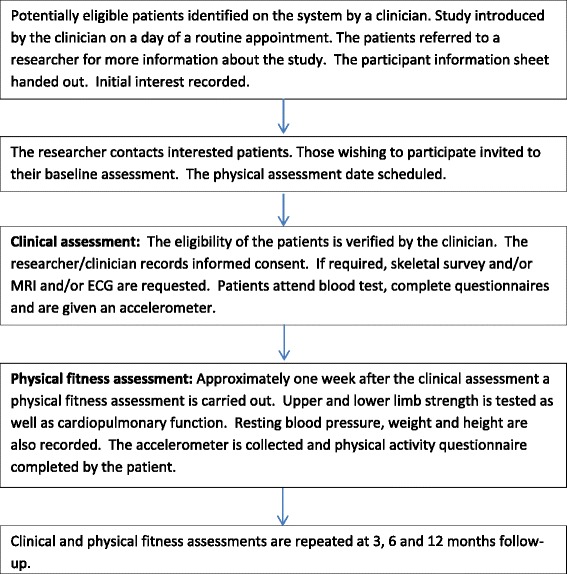
Flow of patients through the lifestyle in myeloma study

The MASCOT cohort was approved by the NRES Committee London – Queen Square (13/LO/1105) and all patients are required to provide informed written consent prior to participation. The study is a prospective cohort study of MM patients in plateau phase to assess physical activity, fatigue and bone health. Clinical markers of health as well as self-reported measures of psychological and physical well-being are also assessed at baseline, 3, 6 and 12 months. At each time point patients complete a clinical assessment, followed by a physical assessment. Flow of patients through the study is shown in Fig. 1.

### Participants

Eligibility criteria are: MM (1) having stable disease (confirmed by a blood test) for at least 6 weeks and either off treatment or on maintenance or consolidation treatment (2) performance status 0–2, as per Eastern Cooperative Oncology Group scoring system (ECOG, [13]) and (3) an ability to provide informed consent. Exclusion criteria are: 1) having spinal instability, 2) having had recent surgery, 3) a poor performance status (ECOG >2), 4) abnormal resting electrocardiogram (ECG) 5) receiving erythroprotein treatment, 6) at risk of pathological fracture (Mirel’s score >7), 7) unstable angina, 8) musculoskeletal disease limiting mobility or 9) cognitive impairment that impedes ability to complete questionnaires. Patients are also ineligible if they are involved in another lifestyle study.

### Recruitment

Recruitment began in June 2014 at the specialist myeloma clinic at University College London Hospital (UCLH) and Royal Free Hospital and is planned to take place over 28 months. If required, patients will also be recruited from St Bartholomew’ Hospital. Approximately 150–200 patients who are in plateau phase are seen annually in each centre. Evidence from a pilot exercise intervention study at UCLH suggested that 80 % of potentially eligible patients would pass eligibility screening [14] and our initial MASCOT screening results have been very similar (>75 % have been eligible). Clinicians identify potential participants in clinic or multidisciplinary team meetings (MDTs) and screen for eligibility, then patients are approached by the research team and provided with information sheets. To date, we have approached 230 eligible patients, of whom 100 have agreed to participate. Once patients are enrolled, the myeloma clinical team are informed and a letter is issued informing their general practitioner (GP).

### Outcome measures

Primary (physical activity, fitness, bone health and fatigue) and secondary outcome measures (well-being, quality of life, diet, self-efficacy, mood, sleep, body composition, muscle strength and endurance) are assessed at baseline, 3 months and 6 months and 12-months.

#### Physical activity and sedentary behavior

Physical activity is assessed using a waist-worn Actigraph wGT3X-BT accelerometer for 7 days at each time point. The Actigraph is a valid and reliable motion sensor that provides an accurate measure of total physical activity and time spent sedentary, in light and in moderate and vigorous physical activity (MVPA). The time spent in activity is expressed in metabolic equivalent units (METs). METs are calculated by dividing the steady state VO2 by 3.5 mL kg^−1^ min^−1^ with the following cut off points <3 METs; 3–5.99 METs; 6.0–8.99 METs; > = 9 METs for sedentary, light, moderate and vigorous activity respectively [15]. The Actigraph is worn in conjunction with Bluetooth® Heart Rate Monitor which records 24 h heart rate. Patients’ participation in PA is also assessed using The Godin Leisure-Time Exercise Questionnaire (GLTEQ, [16] that is widely used in cancer survivors and has acceptable reliability and validity. Patients also complete the GLTEQ via email, telephone or post on a monthly basis throughout the study. The Actigraph is also worn overnight around the wrist to provide a measure of sleep patterns.

#### Cardiorespiratory fitness and strength

Cardiorespiratory fitness is assessed using the MetaLyzer® CPET system (Cortex Biophysik GmbH) and Corival cycle ergometer LODE using VO_2peak_ and anaerobic threshold. Exercise testing is terminated before normal physiologic limitation if the patient shows any of the indications as outlined in the American Thoracic Society/ American College of Chest Physicians (ATS/ACCP) statement on cardiopulmonary exercise testing (CPET, [17]. The level of exertion is assessed using Borg scale [18]. Isometric muscle strength (hand grip strength) is measured with a hand held dynamometer. Three measurements are taken from each arm and the mean of these used. Strength endurance of lower limbs is assessed using a leg press to calculate the maximum load the patient can lift ten times [19]. Lung function is assessed by spirometery and blood pressure taken.

#### Bone health and fatigue

Blood samples are taken at each time point for measurement of markers of bone health (serum levels of OPG & soluble RANKL, serum TRACP-5b, serum TRACP-5b, osteoclacin levels) and vitamin D status. Inflammatory marker CRP is also measured. Fatigue is reported using the 13 item Fatigue Scale of the Functional Assessment of Chronic Illness Therapy (FACIT, [20]. The FACIT is considered appropriate for use with patients with any form of cancer and has been shown to be responsive to change in clinical and observational studies [20].

#### Anthropometrics

Body weight (kg) and percentage body fat and lean mass are assessed using Bioelectrical impedance (TANITA scales model MC-980). Height without shoes is measured using a Leicester height measure and body mass index (BMI) calculated (weight kg/height^2^).

#### Health and lifestyle

Quality of life (QoL) is reported using the Functional Assessment of Cancer Therapy-General (FACT-G), which has subscales for physical, functional, emotional and social/family wellbeing and it has shown positive response to exercise in other cancers [21]. In the current study we are using the emotional and functional well-being subscales of FACT-G. Patients also complete the Hospital and Anxiety and Depressions scale (HADS, [22], widely used to measure emotional distress in cancer patients [23] and report on their sleep patterns using Pittsburgh Sleep Quality Index (PSQI, [24]. In addition, patients complete the Health and Lifestyle Questionnaire [25], which explores diet and assesses desire for lifestyle advice and patients ‘experience of receiving such advice during their care, whether they would like to receive it, at which time-point(s), from whom and in which format(s). Patients’ views on when health behaviour advice should be offered during cancer journey and what factors facilitate and prevent the introduction of behaviour change are also explored in qualitative interviews.

We also assess patients’ confidence in managing their illness and taking part in physical activity (Chronic Disease Self-Efficacy Scales, [26]. Self-efficacy has been found to be correlated with the success in adopting lifestyle changes, including physical activity [27]. Therefore exploring this concept in patients with MM is of interest.

### Predicted attrition rates

Based on attrition rates noted in a previous pilot study in myeloma patients recruited from UCLH [14], in which patients were followed over a period of one year with two follow-up visits in between, we expect that approximately a third will drop out / become ineligible) over the course of the research (as disease relapse is inevitable in MM). Changes from baseline will be assessed for all outcome measures.

### Analyses

Given the exploratory nature of our study and dearth of lifestyle data on which to power our analyses, a pragmatic decision was made to recruit at least 138 participants, to examine the relationship between the outcome variables.

Fatigue and bone health will be analysed using repeated measures (mixed modelling), over the 3 time points (3, 6 and 12 months), after controlling for the baseline measure of fatigue and indicators of bone health. There will also be focus on the effect at 3 months, analysed by linear regression.

Quality of life (FACT-G of the FACIT), and Hospital Anxiety and Depression scale (HADS), will be converted into their standard scores and domains and analysed using linear regression (for the effect by 3 months) and mixed modelling/repeated measures (for all time points). The other outcome measures (physical and exercise capacity endpoints) will also be analysed using the same methods, as will the biochemical markers. Assessments for each outcome will be made to determine whether the data are normally distributed. For outcomes that are not, even after appropriate transformations, non-parametric methods will be used for data analyses at specific time points.

Missing data will be dealt with using methods such as those summarised in http://missingdata.lshtm.ac.uk/talks/RSS_2012_04_18_James_Carpenter.pdf, or chained equations [28].

### Data storage and retention

Data storage and handling will be carried out according to Good Clinical Practice requirements and will be kept for at least 10 years from the date of completion of the project.

### Ethical consideration and dissemination

All participants recruited in this study will provide written informed consent. They will also be reminded that their participation is voluntary and that they have the right to withdraw at any stage without giving a reason, with their usual medical care not being affected in any way.

The results of this study will be disseminated to the academic and clinical audiences in medical, public health and behavioural science meetings and conferences. In addition, the results will be presented in MDT meetings at UCLH, Royal Free and St Bartholomew’s sites. Cancer Research UK will also publish the findings on their website and communicate them to their stakeholders. Moreover, the findings from the study will be presented at national and international haematology meetings, and published in a relevant peer-reviewed journal.

UCL Press office will assist with helping to disseminate the results to the general public and policy makers via press releases.

## Discussion

This study will be the first one to prospectively follow a cohort of patients with Multiple Myeloma in order to document changes in physical activity and sedentary time and fitness, and associations between these and well-being, bone health and fatigue (along with a number of other physical and clinical outcomes). The longitudinal design allows us to investigate and hopefully identify critical points in the disease trajectory at which physical activity may be of optimal benefit to patients with MM.

The study will shed light on what factors determine activity participation in this patient group and will also help to identify the characteristics of patients who are most likely to benefit from taking part in PA. The use of accelerometers will provide further and objective evidence as to the intensity, volume and frequency of PA that would be of optimal benefit to patients with MM.

Finally, the study will address the gap in knowledge about how much and what kind of lifestyle advice patients with myeloma seek and deem to be necessary to improve their cancer management and quality of life after cancer treatment. It is intended that the project determines the scope for future interventions and provides a valuable source of information for lifestyle recommendations for patients with MM.

## Abbreviations

ATS/ACCP, American Thoracic Society/ American College of Chest Physicians; BMI, body mass index; CPET, cardiopulmonary exercise testing; CRF, Cancer-related fatigue; ECG, electrocardiogram; ECOG, Eastern Cooperative Oncology Group scoring system; FACIT, Fatigue Scale of the Functional Assessment of Chronic Illness Therapy; FACT-G, Functional Assessment of Cancer Therapy-General; GLTEQ, The Godin Leisure-Time Exercise Questionnaire; GP, general practitioner; HADS, Hospital and Anxiety and Depressions scale; MASCOT, Myeloma – Advancing Survivor Cancer OuTcomes; MDTs, multidisciplinary team meetings; METS, metabolic equivalent units; MM, Multiple Myeloma; PSQI, Pittsburgh Sleep Quality Index; QoL, Quality of life; UCLH, University College London Hospital
